# Proteomics-based screening and antibiotic resistance assessment of clinical and sub-clinical *Brucella* species: An evolution of brucellosis infection control

**DOI:** 10.1371/journal.pone.0262551

**Published:** 2022-01-13

**Authors:** Ayman Elbehiry, Musaad Aldubaib, Osamah Al Rugaie, Eman Marzouk, Marwan Abaalkhail, Ihab Moussa, Mohamed H. El-Husseiny, Adil Abalkhail, Mohammed Rawway

**Affiliations:** 1 Department of Public Health, College of Public Health and Health Informatics, Qassim University, Al-Bukairiyah, Saudi Arabia; 2 Department of Bacteriology, Mycology and Immunology, Faculty of Veterinary Medicine, University of Sadat City, Sadat City, Egypt; 3 Department of Veterinary Medicine, College of Agriculture and Veterinary Medicine, Qassim University, Qassim, Saudi Arabia; 4 Department of Basic Medical Sciences, College of Medicine and Medical Sciences, Qassim University, Unaizah, Qassim, Saudi Arabia; 5 Department of Clinical Microbiology, King Fahad Medical City, Riyadh, Saudi Arabia; 6 Department of Botany and Microbiology, College of Science, King Saud University, Riyadh, Saudi Arabia; 7 Department of Microbiology, Faculty of Veterinary Medicine, Cairo University, Giza, Egypt; 8 Animal Health Research Institute—Agriculture Research Center, Giza, Egypt; 9 Biology Department, College of Science, Jouf University, Sakaka, Saudi Arabia; 10 Botany and Microbiology Department, Faculty of Science, AL-Azhar University, Assiut, Egypt; Suez Canal University, EGYPT

## Abstract

Brucellae are intracellular sneaky bacteria and they can elude the host’s defensive mechanisms, resulting in therapeutic failure. Therefore, the goal of this investigation was to rapid identification of *Brucella* species collected from animals and humans in Saudi Arabia, as well as to evaluate their resistance to antibiotics. On selective media, 364 animal samples as well as 70 human blood samples were cultured. Serological and biochemical approaches were initially used to identify a total of 25 probable cultured isolates. The proteomics of *Brucella* species were identified using the MALDI Biotyper (MBT) system, which was subsequently verified using real-time polymerase chain reaction (real-time PCR) and microfluidic electrophoresis assays. Both *Brucella melitensis* (*B*. *melitensis*) and *Brucella abortus* (*B*. *abortus*) were tested for antimicrobial susceptibility using Kirby Bauer method and the E-test. In total, 25 samples were positive for Brucella and included 11 *B*. *melitensis* and 14 *B*. *abortus* isolates. Twenty-two out of 25 (88%) and 24/25 (96%) of *Brucella* strains were recognized through the Vitek 2 Compact system. While MBT was magnificently identified 100% of the strains at the species level with a score value more than or equal to 2.00. Trimethoprim-sulfamethoxazole, rifampin, ampicillin-sulbactam, and ampicillin resistance in *B*. *melitensis* was 36.36%, 31.82%, 27.27%, and 22.70%, respectively. Rifampin, trimethoprim-sulfamethoxazole, ampicillin, and ampicillin-sulbactam resistance was found in 35.71%, 32.14%, 32.14%, and 28.57% of *B*. *abortus* isolates, correspondingly. MBT confirmed by microfluidic electrophoresis is a successful approach for identifying *Brucella* species at the species level. The resistance of *B*. *melitensis* and *B*. *abortus* to various antibiotics should be investigated in future studies.

## Introduction

Brucellosis is a prevalent zoonotic disease that continues to be community health obstacles around the world [1]. Brucellosis has remained endemic in many parts of the Universe till now, with an estimated 500 thousand new cases each year [2, 3]. *Brucella* is an intracellular gram-negative bacterium that harms a variety of domesticated animals, comprising cattle, sheep, goats, and camels [4, 5]. *B*. *melitensis*, which was first isolated from sheep, goats, and camels; *B*. *abortus* of cows; *Brucella suis* (*B*. *suis*) of pigs; and *Brucella canis* (*B*. *canis*) of canines are all virulent to humans [4].

Different species of *Brucella* are known to have varying degrees of pathogenicity. *B*. *melitensis* is one of the most prevalent aggressive and pathogenic species of *Brucella*, causing the most serious sickness [6]. *B*. *abortus*, on the other hand, is the least pathogenic species and causes the sickness to be mild. As a result, *B*. *melitensis* is often regarded as the primary cause of brucellosis in humans around the world. Despite being under control in several affluent nations, the disease stills prevalent in the Kingdom of Saudi Arabia, where the disease has a nationwide sero-prevalence of about 15% [7]. This disease is brought into Saudi Arabia via the unrestricted importation of various animals that have not been thoroughly checked for the disease [7, 8].

Nevertheless, isolation of *Brucella* from various cultures and identification by traditional techniques are considered the standard methods for identification brucellosis in both humans and different animal species, these methods remains unsafe, laborious, and expensive [9, 10]. Several serological screening tests such as Rose Bengal Plate Test (RBPT), Serum Agglutination Test (SAT), Enzyme Linked Immunosorbent Assay (ELISA) and Complement Fixation Test (CFT) are still applied for the recognition of Brucella antibodies [11]. The World Health Organization (OIE) stated that the CFT is the most widely accepted test in the world [12]. However, this test has many disadvantages such as time-consuming and hard to standardize [13]. The above mentioned tests did not have the ability to differentiate between antibodies which have been formed following vaccination and those resulted after infection [14].

Molecular diagnostic techniques are also crucial tools in the detection of Brucellosis. Conventional PCR techniques using primers such as 16S rRNA [15], the 16S-23S intergenic spacer region [16], omp2 [17] and bcsp31 [18], have been well-known for discovery of Brucellae gene sequences. These techniques were improved for detection of Brucellae in various biomedical samples. Leyla et al. [19] confirmed that standard PCR is considered a respectable method to identify DNA of *Brucella* species isolated from clinical samples, however, Romero and coworkers established that PCR had minor accuracy than the other traditional tools [20].

Although genetic analyses are still used as truthful methods for diagnosis of various types of microorganisms, their application is time consuming, and their cost may be relatively high. Because brucellosis represents one of major laboratory-acquired diseases [21], the fast recognition of *Brucella* species is essential to protect the handlers of microbiology laboratories [22]. Moreover, the Centers for Disease Control and Prevention ordered brucellae as one of the bioterrorism agents; consequently, early discovery of this bacterium is very significant to reduce its risk. Consequently, there is an essential requirement for fast, low-cost, less-skilled laboratory personnel and precise method for recognizing the various microbes causing infectious and non-infectious diseases.

Therefore, an innovative skill for precise and rapid categorization of various microorganisms is an actual phase toward an appropriate method for handling of contagious infections in medical and veterinary diagnostics [23, 24]. Currently, the furthermost appropriate technique for recognition of various pathogens is based on mass spectral identification with MALDI Biotyper (MBT) [25]. By using of this technique, the various bacteria and fungi are identified by corresponding the obtained mass spectra with the mass spectra deposited in the reference library. In recent times, a reference library to identify various types of Brucella at the genus and species levels was carried out using 12 Brucella strains [26].

From this perspective view, MBT can be used to diagnose brucellosis in humans and animals in a timely and accurate manner [27]. MBT has many advantages than the other techniques such as reducing the danger of laboratory infections and the time used to detect the infection [26, 28, 29]. Proteomic analysis of mass peak intensities permits the actual documentation to the species level of bacteria [25]. The main idea of this technology is mainly depended on ionization of the microbial proteins by laser shots and the creation of different peak intensities known as spectra. Through the properties of a spectra database kept in the library of MBT device, the associated software scans for matching with the microbial species, based on a consistent list between both spectra [30, 31].

Most antibiotics are unable to penetrate Brucellae because of their intracellular position in reticuloendothelial cells and their preference sites (e.g., bone). To treat brucellosis, antimicrobial regimens containing quinolones, doxycycline, rifampicin, streptomycin, and aminoglycosides are being used alone or in combination [32, 33]. Failure of treatment is common, and there have been lots of reports of brucellosis relapses after treatment, varying from 5% to 15% in mild cases [34]. Emergence of multidrug-resistant in *Brucella* has recently emerged in brucellosis-endemic regions worldwide (for example, Malaysia, Egypt, Qatar, and China) [34].

Multidrug resistance bacteria arise as a result of incorrect antimicrobial usage [33, 35, 36]. Antibiotics used to encourage growth or as a primary prevention in domesticated animals contribute to the enhancement of bacterial resistance and have a significant impact in their dissemination throughout the food supply chain [37]. Resistance to antibiotics in zoonotic infections is also a concern, as it limits disease treatment choices in public health and animal contexts [38]. None of the research that are available show antimicrobial susceptibility profiles of Brucella isolates from Saudi livestock and humans. Multidrug resistance has been increased all over the world that is considered a public health threat. Several recent investigations reported the emergence of multidrug-resistant bacterial pathogens from different origins including humans, birds, cattle, and fish that increase the need for routine application of the antimicrobial susceptibility testing to detect the antibiotic of choice as well as the screening of the emerging MDR strains [39–45].

Drug sensitivity screening is the approach for effective brucellosis management and treatment [46]. Microdilution and/or E-test procedures are used to calculate the minimum inhibitory concentrations (MICs) of antimicrobial drugs [46, 47]. Our research aimed to use serological tests, the Vitek 2 compact System, and MBT with real-time PCR confirmation to rapidly and precisely identify *Brucella* species isolated from sheep, goats, and humans with a history of brucellosis. A Kirby Bauer method and E-test are also used to test their resistance to various antibacterial drugs.

## Material and methods

### Ethical statement

Because there were no human or animal participants in this study, it did not involve ethical approval or written permission. Only bacterial cultures from regular medical testing or strain collections have been used, not primary human or animal samples. All of the clinical strains used in this study came from regular diagnostic tests. As a result, there was no attempt to get patient or animal samples for the investigation.

### Sample collection and isolation of Brucella species

A total of 434 samples were obtained from humans and various animal herds in the Al-Qassim region of Saudi Arabia with a history of habitual abortion. Three hundred and sixty-four samples were collected from animal sources as follow: 124 milk samples (80 from cows and 44 from goats), 120 blood and serum samples (80 from cows and 40 from goat) and 120 vaginal swab samples (80 from cows and 40 from goats). Seventy from each human blood and serum samples were also collected from people suffering from hyperthermia who were in direct contact with suspected animals. All samples were collected between April to September, 2021.

In the Al-Qassim region of Saudi Arabia’s central plateau, 15 ml of each milk and blood sample, along with vaginal swab samples, were collected from cows and goat farms with a history of habitual abortion. Biosafety level two (BSL2) was used for all bacteriological samples with high personal percussions as formerly considered. Concisely, the milk samples were rotated at 6000 rpm for 10 min to concentrate the organism under hygienic procedures that decrease the danger of aerosol infection to the technicians, and the sediment (cream) was inoculated onto specific media (*Brucella* Selective Agar) supplemented with antibiotic as formerly described [48], after that the cultured plates were examined for the growth of Brucella species on the fourth day and thereafter day-to-day for 2–4 weeks at 37°C in the presence or absence of CO_2_ (5–10%).The suspected colonies of *Brucella* were sub-cultured till appearance of typical round, glistening, pinpoint and honey drop-like appearance. Furthermore, 6 isolates of *B*. *melitensis* were collected from microbiological laboratories in a number of hospitals, which took precautions while extracting blood and serum samples from 70 febrile patients based on their history, which involved animal contact, unpasteurized milk consumption, and clinical signs for example fever, night sweetening, low back pain, and severe joint pains. Subsequent biochemical analysis of all *Brucella* isolates were carried out with Vitek® 2 Compact System.

### Biochemical identification of the recovered isolates

The suspected isolates that illustrated gram negative short coccobacilli from oxidase and catalase positive small colonies were examined for production of urease and H_2_S. On Columbia agar (Sigma-Aldrich, Saint Louis, USA), the strains were recognized by the Vitek 2 device (bioMérieux SA F-69280 Marcy l’Etoile France), via GN cards (bioMérieux SA F-69280 Marcy l’Etoile France) as indicated in the company’s guidelines. Concisely, the bacterial suspension was prepared and balanced by McFarland standards (0.5 to 0.63). Vitek®2 cards were inoculated, and the cards were then submitted to the machine to for proper identification.

### Serological identification of the recovered *Brucella* species

#### Rose Bengal Test (rapid slide agglutination antigen)

The Rose Bengal Test (RBT) was applied to discriminate antibodies against *B*. *abortus* and *B*. *melitensis* strains in animal and human serum samples [49]. This test depends mainly on reaction between the bacterial suspension and immunoglobulins (G & M antibodies) in clinical and sub-clinical infections. The test is carried out by examination of the buffered suspension (pH 3.6) of *B*. *abortus* strain colored with Rose Bengal against unrecognized sera. The test kits including Rose Bengal Antigen, positive and negative control were purchased from Linear Chemicals, Spain. In brief, the antigen vial was gently re-suspended several times. Fifty μL (one drop) of each unknown serum was placed into one circle of the card. Subsequently, one drop of positive control serum and another drop of negative control serum were dispensed into two separated circles. One drop of Rose Bengal Antigen was then added to each circle. The contents of each circle were properly mixed with one-use stirrer and the slide was manually rotated for approximately 4 minutes. Any degree of agglutination was directly observed under light source and then interpreting the results.

#### Complement-Enzyme Linked Immuno Sorbent Assay (cELISA)

The cELISA was achieved and results were recorded [50] as described by the guidelines of the manufacturing via Svanovir™ Brucella-Ab cELISA kit (Svanovia Biotech AB Uppsala, Sweden).

### Protein analysis using MALDI Biotyper (MBT)

The MBT equipment, which was obtained from Bruker Daltonik in Bremen, Germany, was utilized to identify *Brucella* species isolated from humans and other animal species (cows and goats) suffering from clinical and sub-clinical Brucellosis quickly and precisely. FlexControl (Flex Series version 1.3) and Compass software were used to investigate all isolates [25].

#### Preparation of samples via ethanol/formic acid extraction procedure

All MBT samples were made by cultivating on modified Farrell’s serum dextrose agar (Sigma-Aldrich, USA) and then incubating at 37°C for 3–7 days. The extraction of ethanol and formic acid was carried out according to Bruker Daltonics’ guidelines. In brief, 1–2 new colonies were placed onto a clean Eppendorf tube and carefully mixed in 300 μl of sterilized water for each sample. Then, nine hundred μl of pure ethanol were added. The contents were vortexed carefully, and the tubes were correctly mixed for two minutes at 13,000 rpm, with the supernatant decanted and the pellet air-dried. After that, ten μl of the pellet were thoroughly rotated with fifty μl of 70% formic acid, followed by an equal amount of acetonitrile. One μl of the supernatant was put onto a stainless steel plate and left to dry at 25°C after another 2 minutes of spin at 13,000 rpm. As a result, each isolate was covered with one μl of cyano-4 hydroxycinnamic acid (HCCA) matrix solution. The MBT target plate was then loaded into the MBT system for automatic data collection and analysis. All samples were run twice to ensure good detection. The positive control (*Escherichia coli*) was made by inoculating 50 μl of the standard solvent solution onto the in-vitro diagnostic product (IVD) BTS pellet and melting it repeatedly at 27°C. The solution of Bacterial Test Standard (BTS) was then dissolved for approximately five min at the same manner for several times and then rotated properly at 13, 000 rpm for two minutes. Lastly, the supernatant (5 μl) was moved into screw cap tubes and is kept at –20°C for additional examinations.

#### Data analysis in MBT

By comparing the unidentified spectrum to the known spectrum preserved in the reference library, the log score of an unnamed spectrum in the range of 0 to 3 was calculated. In the ranges of 2 to 2.29 and 1.700 to 1.999, the species and genus levels were predicted. Misidentification, on the other hand, was carried out when the score value fluctuated between 0.00 and 1.69. The m/z range of two thousand Da to twenty thousand Da was used to evaluate the various spectra created by the Compass Software. A dendrogram based on the reference library was built from the minimum spanning tree (MSP) data set, which includes over 7,000 different species of microorganisms.

### SYBR green real time PCR assay

#### DNA extraction

The field isolates’ DNA was extracted using the QuickGene-810 (AutoGen, Japan) and the QuickGene DNA tissue kit S (DT-S) as stated by the producer’s guidelines. A Spectrophotometer (NanoDropTM 2000) obtained from Thermo Scientific was used to determine the degree of purity of the isolated DNA.

#### qPCR assay

As a MBT confirmatory procedure, the Fast 7500 Real-Time PCR System (Applied Biosystems, USA) was used. This method was used to distinguish between *B*. *melitensis* and *B*. *abortus*. *BMEII0466* gene for *B*. *melitensis* and *BruAb2 0168* gene for *B*. *abortus* were selected for the manufacture of species-specific primers (Table 1). The reaction mix was made up of 15 μl (10 μl of oasigTM or Precision PLUSTM 2 qPCR Master Mix, 1 μl of primer/probe mix, and 4 μl of RNAse/DNAse-free water). After that, 15 μl of this combination was placed into each well, followed by 5 μl of DNA template, while the negative control wells received 5 μl of RNAse/DNase free water. As a result, each well’s final volume was 20 μl. For infection interpretation, the amplification findings were plotted against the cycle number as Delta Rn (Rn).

**Table 1:** Oligonucleotide sequences used in the current study.

Species	Target sequence	Forward (F) primer/reverse (R) primer (5′→3′)		Base pair (bp) size	Reference
*B*. *melitensis*	*BMEII0466*	F	TCGCATCGGCAGTTTCAA	112	[80]
		R	CCAGCTTTTGGCCTTTTCC		
*B*. *abortus*	*BruAb2_0168*	F	GCACACTCACCTTCCACAACAA	222	
		R	CCCCGTTCTGCACCAGACT		

### Analysis of PCR products by LabChip GX Touch 24

Electrophoresis for PCR products will be accomplished by LabChip GXII Touch 24 instrument (PerkinElmer, USA). DNA 1K Assay Quick was applied for chip and processing of samples. In brief, the chip and reagents were prepared by equilibration for 2 min at room temperature. The Dye Concentrate (blue cap) was properly melted and vortexed. For four Low-throughput chip preparations, one vial of DNA HiSens/NGS3K Gel Matrix (red cap) was advised. Gel-Dye was by transferring 13 μl of DNA Dye Concentrate into one vial of DNA Gel Matrix and then was vortexed properly. The mixture was transferred into two spin filters and centrifugation was carried out at 9200 rcf for 10 minutes. Each active well (1 to 10) was rinsed aspirated with purified water. Fifty μl of gel-dye was then added by reverse pipetting method to chip well 3, 7, 8 and 10 and 50 μl DNA Marker was added to chip well 4 and finally, the chip was inserted in the device.

DNA Sample, Ladder and Buffer preparation was carried out as follow: Firstly, 14 μl DNA ladder and 106 μl of DNA sample buffer were added to the provided 0.2 ml ladder tube. The ladder tube was inserted into the ladder hole on the LabChip holder. Then 750 μl (150 μl DNA sample buffer plus 600 μl water) of DNA sample buffer was added to the buffer tube (0.75 ml) delivered with the reagent kit. The buffer tube was then inserted into the buffer slot on the LabChip device. Finally, 40 μl of each DNA sample was transferred into the microchannels and analyzed by electrophoresis. The DNA samples from 25–12,000 bps was rapidly characterized after 30–60 seconds.

### Susceptibility of *Brucella* species to various antibiotics

A total of 25 isolates of Brucella species (11 *B*. *melitensis* and 14 *B*. *abortus*), were included in the current study. A method described by Magiorakos et al. [51] was used to classify the tested isolates into 18 multidrug-resistant (MDR) strains and 7 extensively drug-resistant (XDR) strains.

The E-test (MIC Test Strip, Fisher Scientific) and Kirby-Bauer techniques were used to assess Brucella species vulnerability and resistance to 12 antimicrobial drugs (Table 2) routinely used to treat human and animal brucellosis. The two tests were undertaken in accordance with the CLSI requirements [51]. The MICs of field isolates to 6 classes of antibiotics including penicillins [ampicillin (AMP, 0.016–256 μg/mL), ampicillin-sulbactam (AMS, 0.016–256 μg/mL)], cephalosporins [cefuroxime (CXM, 0.016–256 μg/mL)], tetracyclines [tetracycline (TE, 0.016–256 μg/mL), doxycycline (DXT, 0.016–256 μg/mL)], fluoroquinolones [ciprofloxacin (CIP, 0.002–32 μg/mL), levofloxacin (LEV, 0.002–32 μg/mL)], sulfonamides [trimethoprim- sulfamethoxazole (SXT, 0.002–32 μg/mL), chloramphenicol (C, 0.016–256 μg/mL)], rifamycins [rifampin (RIF, 0.016–256 μg/ml)], and aminoglycosides [gentamycin (CN, 0.064–1024 μg/mL), streptomycin (S, 0.016–256 μg/mL)] were measured using MIC Test Strip purchased from Fisher Scientific, US.

**Table 2:** Zone diameter and MIC breakpoints for *Haemophilus* spp. as an alternative for *Brucella* species [52].

Antibiotic used	Disc content in μg	Zone diameter breakpoints in mm			MIC breakpoints in μg/ml		
		S	I	R	S	I	R
Ampicillin	10	≥22	19–21	≤18	≤1	2	≥4
Ampicillin-sulbactam	10/10	≥20	-	≤19	≤ 2/1	–	≥ 4/2
Cefuroxime	30	≥20	17–19	≤16	≤4	8	≥16
Tetracycline	30	≥29	26–28	≤25	≤2	4	≥8
Doxycycline	30	≥48	32–47	≤31	≤1	-	-
Ciprofloxacin	5	≥ 21	-	≤20	≤1	-	-
levofloxacin	5	≥ 17	18–19	≤20	≤2	-	-
Trimethoprim- sulfamethoxazole	1.25/23.75	≥16	11–15	≤10	≤ 0.5/9.5	1/19–2/38	≥ 4/76
Chloramphenicol	30	≥29	26–28	≤25	≤2	4	≥8
Rifampin	5	≥20	17–19	≤16	≤1	2	≥4
Gentamycin	10	≥45	23–44	≤22	≤4	-	-
Streptomycin	10	≥36	20–35	≤19	≤8	-	-

Measuring zones for ampicillin (10 μg), ampicillin-sulbactam (10/10 μg), cefuroxime (30 μg), tetracycline (30 μg), doxycycline (30 μg), ciprofloxacin (5 μg), levofloxacin (5 μg), trimethoprim- sulfamethoxazole (1.25/23.75 μg), chloramphenicol (30 μg), rifampin (5 μg), gentamycin (10 μg), and streptomycin (10 μg) was established by Kirby-Bauer method and automatically identified by ProtoCOL 3 Plus (Synbiosis, Cambridge, United Kingdom). Preparation of bacterial suspension for each isolate was performed using pure colonies and the McFarland Standard 0.5 (Thermo Fisher Scientific Inc., US) was applied for adjustment of the bacterial turbidity. The suspension of each isolate was then distributed onto Muller-Hinton agar plates (IndiaMart, India) enhanced with sheep blood (5%) and in the presence of 10% CO_2_, all plates were incubated at 37°C for two successive days. The breakpoints of *Brucella* species have been detected against the antimicrobial agents under study, based on the procedures for slow-growing bacteria (*Haemophilus* spp.) as formerly described [52]. All antibiotics were tested two times to confirm the results. *B*. *melitensis* biotype 1 (strain 16M / ATCC 23456) and *Haemophilus influenzae* ATCC^®^ 10211™, were applied as reference strains throughout the current investigation.

## Results

### Isolation of *Brucella* species and colony physiognomies

Initially, the growth of bacterial colonies was illustrated as early as 72 h on a specific media (*Brucella* selective agar) and the majority of isolates were noticed following four consecutive days of incubation at 37°C with absence of CO_2_. Under microscope, the grown colonies exhibited typical honey-like appearance which characterized by small, smooth, round and pin-point colonies. Modified Ziehl-Neelsen (MZN) stain was applied for all isolates to demonstrate their cellular characteristics (e.g. gram negative small coccobacilli arranged singly and in pairs). According to our findings in Table 3, 14 isolates were isolated from 240 cow samples as follow: 5 strains from 80 milk samples, 3 strains from blood samples and 6 strains from vaginal swab samples whereas, 5 strains were recovered from 44 goat samples (1 strain from milk, 2 from blood & 2 from vaginal swab samples). From 70 human blood samples, 6 strains were detected.

**Table 3:** Different types of samples used in the study and *Brucella* isolates.

Species	Type of sample						Total	
	Milk		Blood		Vaginal swab			
	Sample cultured	Isolates	Sample cultured	Isolates	Sample cultured	Isolates	Total samples cultured	Total isolates
**Cattle**	80	5	80	3	80	6	240	14
**Goat**	44	1	40	2	40	2	124	5
**Human**	0	0	70	6	0	0	70	6

### Biochemical and serum analyses

Twenty-five isolates were biochemically identified by the Vitek 2 ID-GN card. Based on our findings, the examined strains were recognized as 11 *B*. *melitensis* and 14 *B*. *abortus*. This test was carried out in around 7–8 hours for majority of the strains as indicated previously by Pappas et al. (2006), The minimum period used to check the isolates was 6 hours (3 isolates), while the longest period was 10.40 hours (2 isolates). One hundred and ninety serum samples collected from human (n = 70), cow (n = 80) and goat (n = 40) were examined by RBT and cELISA technique and the interpreting results indicated that the positive samples for RBT were 10% (7/70), 3.75% (3/80) and 5% (2/40), for human, cow and goat samples, respectively. All positive samples for RBT were confirmed cELISA except one human serum samples gave negative results.

### Protein fingerprinting of *Brucella* species

**Fig 1:**
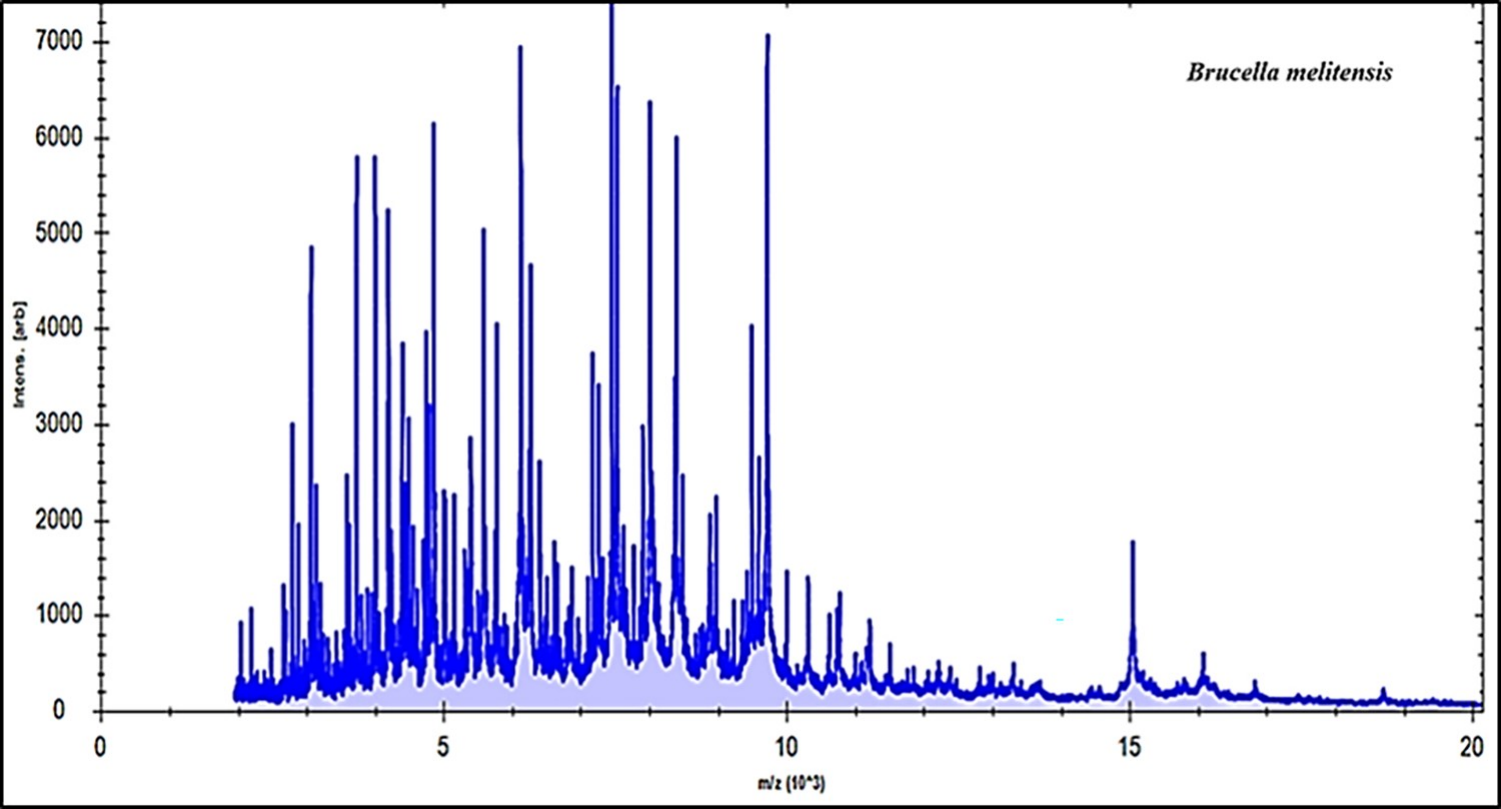
Protein patterns of Brucella melitensis strain.

**Fig 2:**
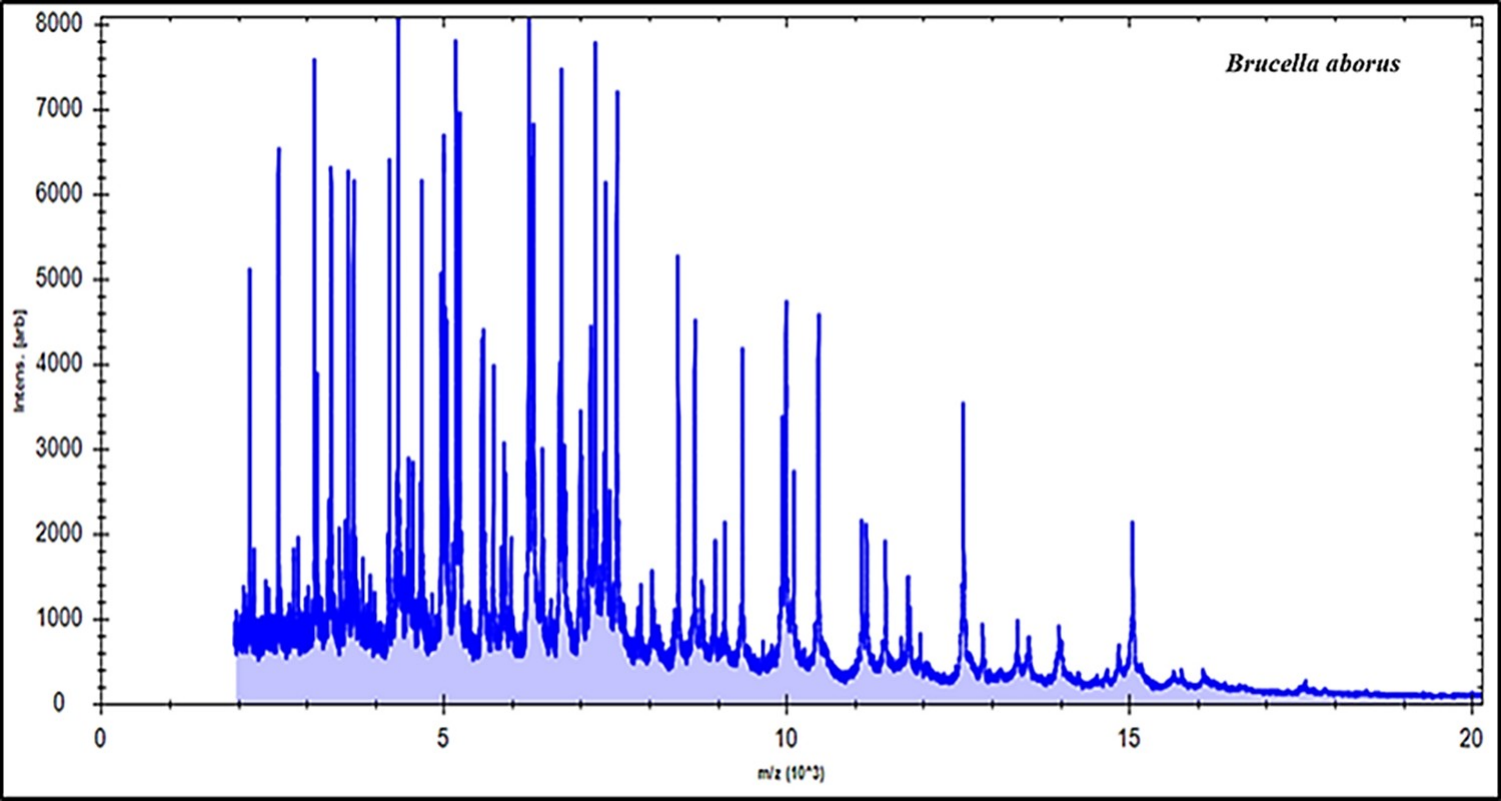
Protein patterns of field *Brucella abortus* strain.

In the current investigation, 25 cultured strains were examined by MBT device, and the spectra of the field isolates were correlated to the reference spectra. Based on our findings, MBT was able to identify all cultured isolates as *Brucella* species (11 *B*. *melitensis* and 14 *B*. *abortus*) by 100%. Examining these results illustrated that about twenty prominent ion peaks were identified in the original bands from the region extended from 2000 to 15,000 Daltons (Da) (Figs 1 & 2). All tested strains were appropriately recognized as 14 *B*. *abortus* strains recovered from cows and 11 *B*. *melitensis* recovered from human (6 strains) and goat (5 strains).

As can be seen in Table 4, analysis of logarithmic score value demonstrates that 5/14 (35.71%) *B*. *abortus* and 3/11 (27.27%) of *B*. *melitensis*, were identified score value between 2.3 and 3.0. Similarly; 8/14 (57.14%) *B*. *abortus* and 8/11 (72.73%) *B*. *melitensis* were properly identified, with log values ranging from 2.0 to 2.29. However, we found that one strain (3.57%) of *B*. *abortus* was identified at the genus level at less than 2 scoring value.

**Table 4:** Logarithmic score values for *B*. *melitensis* and *B*. *abortus* strains isolated from human blood samples and milk and vaginal swab samples of animals by MBT.

Brucella spp.	Total number	Score value of identification							
		2.3–3		2–2.29		1.7–1.99		0–1.69	
		No.	%	No.	%	No.	%	No.	%
*B*. *melitensis*	11	3	27.27	8	72.73	0	0.00	0	0.00
*B*. *abortus*	14	5	35.71	8	57.14	1	3.57	0	0.00

**Fig 3:**
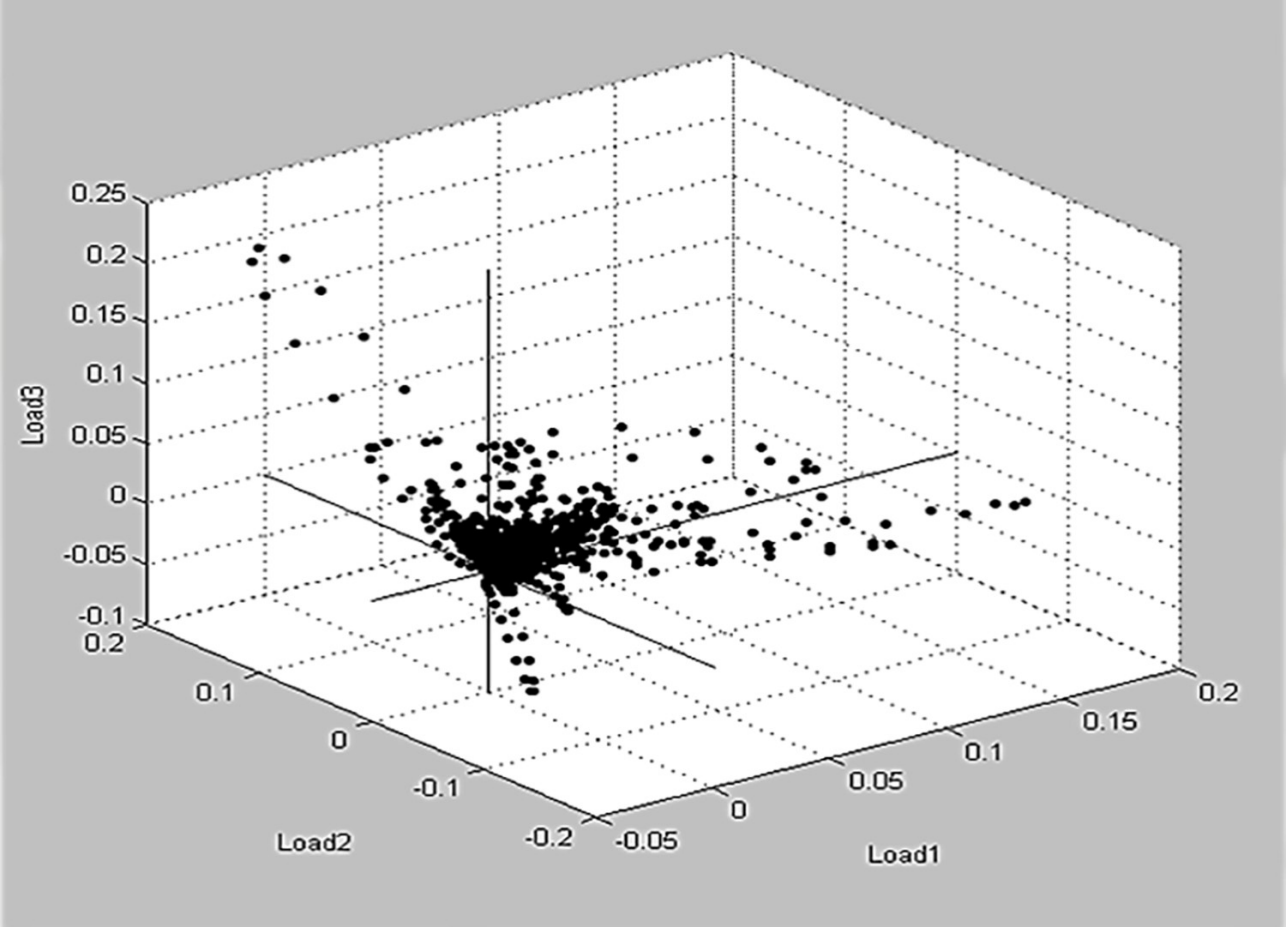
Principal component analysis developed a 3D loading image that shows many spectra for 11 *Brucella melitensis* and 14 *Brucella abortus* strains. The force value of the peaks was represented by each dot. The peaks were adjusted in accordance with the loading value, which corresponded to loading 1, loading 2, and loading 3 modes.

Principal Component Analysis (PCA) was created by MBT Compass IVD software as a supplementary statistical tool to detect the resemblances and variances among the spectral proteins of Brucella species. As shown in Fig 3, three‐dimensional (3d) image of PCA illustrated numerous protein spectra. Detection of each peak was demonstrated by calculation of the three loading standards (Loading 1, Loading 2, and Loading 3). In our investigation, the majority of peaks verified in the MBT Compass IVD software were assessed statistically by PCA, which was capable of separating the *Brucella* species as *B*. *melitensis* and *B*. *abortus*.

### Molecular identification of *Brucella* species

SYBR® Green qPCR was applied to approve the results of MBT. The target sequences of *BMEII0466* and *BruAb2_0168* were considered to recognize *B*. *melitensis* and *B*. *abortus*, respectively. Amplification of PCR with these target sequences produced replication events of the predictable base pairs. Every genome was magnified individually, and the size of each probable product was long-established. Based on our findings, *BMEII0466* and *BruAb2_0168* were detected in 11 *B*. *melitensis* and 14 *B*. *abortus* strains, correspondingly. The products of qPCR were run through LabChip GXII Automated electrophoresis device to analyze the size of both genes and the results indicated that the sizes were 112 bp for *B*. *melitensis* and 222 bp for *B*. *abortus*. By comparing the results of MBT with the qPCR, it was found that there is complete agreement; consequently, the qPCR is used as a confirmatory method of MBT.

### Susceptibility of *B*. *melitensis* and *B*. *abortus* strains to antimicrobial agents

Based on the measurements of MIC for all tested antibiotics as can be seen in Table 5, it was appeared that all strains were vulnerable to ceftriaxone (MIC_90_ = 2 μg/ml for *B*. *melitensis* and MIC_90_ = 0.75 μg/ml for *B*. *abortus*), ciprofloxacin (MIC_90_ = 0.125 μg/ml for *B*. *melitensis* and MIC_90_ = 0.094 μg/ml for *B*. *abortus*), levofloxacin (MIC_90_ = 1 μg/ml for *B*. *melite nsis* and MIC_90_ = 0.75 μg/ml for *B*. *abortus*). According to the results recorded in Table 6 and Table 7, 36.36%, 31.82%, 27.27% and 22.70% of *B*. *melitensis* strains were resistant to trimethoprim-sulfamethoxazole (MIC_90_ = 0.125 μg/ml), rifampin (MIC_90_ = 4 μg/ml), ampicillin-sulbactam (MIC_90_ = 1.5 μg/ml) and ampicillin (MIC_90_ = 4 μg/ml), respectively. Likewise, 35.71%, 32.14%, 32.14, and 28.57% (Table 7) of *B*. *abortus* isolates were resistant rifampin (MIC_90_ = 6 μg/ml), trimethoprim-sulfamethoxazole (MIC_90_ = 0.38 μg/ml), ampicillin (MIC_90_ = 3 μg/ml) and ampicillin-sulbactam (MIC_90_ = 2 μg/ml), respectively.

**Table 5:** MBT and molecular identification, as well as culturing of *B*. *melitensis* and *B*. *abortus* isolated from human animal species in Saudi Arabia.

ID of sample	Species	Sample’s origin	Sample’s type	Selective culture media	MBT identification		Real time PCR
					Species	Score value	
CM-1	Cattle	Bukiryah	Milk	Positive	*B*. *abortus*	2.12	*B*. *abortus*
CM-2	Cattle	Bukiryah	Milk	Positive	*B*. *abortus*	2.31	*B*. *abortus*
CM-3	Cattle	Unayzah	Milk	Positive	*B*. *abortus*	2.16	*B*. *abortus*
CM-4	Cattle	Unayzah	Milk	Positive	*B*. *abortus*	2.43	*B*. *abortus*
CM-5	Cattle	Ar Rass	Milk	Positive	*B*. *abortus*	2.08	*B*. *abortus*
CB-6	Cattle	Ar Rass	Blood	Positive	*B*. *abortus*	2.29	*B*. *abortus*
CB-7	Cattle	Ar Rass	Blood	Positive	*B*. *abortus*	2.30	*B*. *abortus*
CB-8	Cattle	Ar Rass	Blood	Positive	*B*. *abortus*	2.22	*B*. *abortus*
CV-9	Cattle	Bukiryah	Vagina	Positive	*B*. *abortus*	2.41	*B*. *abortus*
CV-10	Cattle	Buraydah	Vagina	Positive	*B*. *abortus*	2.14	*B*. *abortus*
CV-11	Cattle	Unayzah	Vagina	Positive/Negative	*Brucella spp*.	1.98	*B*. *abortus*
CV-12	Cattle	Buraydah	Vagina	Positive	*B*. *abortus*	2.18	*B*. *abortus*
CV-13	Cattle	Unayzah	Vagina	Positive	*B*. *abortus*	2.39	*B*. *abortus*
CV-14	Cattle	Unayzah	Vagina	Positive	*B*. *abortus*	2.21	*B*. *abortus*
GM-15	Goat	Unayzah	Milk	Positive	*B*. *melitensis*	2.24	*B*. *melitensis*
GM-16	Goat	Ar Rass	Blood	Positive	*B*. *melitensis*	2.32	*B*. *melitensis*
GM-17	Goat	Ar Rass	Blood	Positive	*B*. *melitensis*	2.00	*B*. *melitensis*
GM-18	Goat	Buraydah	Vagina	Positive	*B*. *melitensis*	2.23	*B*. *melitensis*
GM-19	Goat	Buraydah	Vagina	Positive	*B*. *melitensis*	2.15	*B*. *melitensis*
HB-20	Human	Buraydah	Blood	Positive	*B*. *melitensis*	2.41	*B*. *melitensis*
HB-21	Human	Buraydah	Blood	Positive	*B*. *melitensis*	2.07	*B*. *melitensis*
HB-22	Human	Buraydah	Blood	Positive	*B*. *melitensis*	2.36	*B*. *melitensis*
HB-23	Human	Buraydah	Blood	Positive	*B*. *melitensis*	2.18	*B*. *melitensis*
HB-24	Human	Riyadh	Blood	Positive	*B*. *melitensis*	2.10	*B*. *melitensis*
HB-25	Human	Riyadh	Blood	Positive	*B*. *melitensis*	2.20	*B*. *melitensis*

**Table 6:** The MIC values of antimicrobial agents against 25 *Brucella* strains (11 *B*. *melitensis* and 14 *B*. *abortus*).

Antimicrobial agent	Conc. in μg/ml	Range of MIC		MIC_50_ in μg/ml		MIC_90_ in μg/ml	
		*B*. *melitensis*	*B*. *abortus*	*B*. *melitensis*	*B*. *abortus*	*B*. *melitensis*	*B*. *abortus*
AMP	0.016–256	0.25–6	0.19–4	0.38	1.5	4	2
AMS	0.016–256	0.125–6	0.094–6	1	0.75	1.5	3
CXM	0.016–256	0.75–8	0.5–6	1.5	1.5	2	0.75
TE	0.016–256	0.064–8	0.094–8	0.125	0.19	0.25	0.75
DXT	0.016–256	0.032–4	0.047–4	0.094	0.125	0.19	0.25
CIP	0.002–32	0.023–0.19	0.023–0.25	0.047	0.19	0.125	0.094
LEV	0.002–32	0.032–1.5	0.094–1	0.75	0.50	1	0.75
SXT	0.002–32	0.012–4	0.016–0.25	0.047	0.19	0.125	0.38
C	0.016–256	0.032–0.75	0.023–0.5	0.064	0.094	0.25	0.19
RIF	0.016–256	0.125–6	0.094–12	0.19	4	4	6
CN	0.064–1024	0.38–6	0.50–6	0.75	0.75	1	0.50
S	0.016–256	0.023–8	0.032–8	0.125	0.19	0.38	0.75

AMP = ampicillin; AMS = ampicillin-sulbactam; CXM = cefuroxime; TE = tetracycline; DXT = doxycycline; CIP = ciprofloxacin; LEV = levofloxacin, SXT = trimethoprim- sulfamethoxazole; C = chloramphenicol; RIF = rifampin; CN = gentamycin; S = streptomycin

**Table 7:** Kirby Bauer method for determining antibiotic sensitivity of *B*. *melitensis* and *B*. *abortus* isolates.

Antimicrobial agent	Conc. in μg/ml	Range in mm		Degree of susceptibility											
				B. melitensis (n = 11)						B. abortus (n = 14)					
		B. melitensis	B. abortus	S		I		R		S		I		R	
				No.	%	No.	%	No.	%	No.	%	No.	%	No.	%
AMP	10	13–35	16–34	17	77.30	0	0	5	22.7	19	67.86	0	0	9	32.14
AMS	10/10	14–30	12–33	16	72.73	0	0	6	27.27	20	71.43	0	0	8	28.57
CXM	30	24–37	22–41	22	100.0	0	0	0	0.00	28	100.0	0	0	0	0.00
TE	30	25–40	25–38	20	90.91	0	0	2	9.10	25	89.29	0	0	3	10.71
DXT	30	26–52	27–51	20	90.10	0	0	2	9.10	26	92.86	0	0	2	7.14
CIP	5	22–37	24–40	22	100.0	0	0	0	0.00	28	100.0	0	0	0	0.00
LEV	5	18–29	17–32	22	100.0	0	0	0	0.00	28	100.0	0	0	0	0.00
SXT	1.25/23.75	7–23	6–24	14	63.64	0	0	8	36.36	17	60.71	2	7.14	9	32.14
C	30	25–42	26–38	19	86.36	0	0	3	13.66	26	92.86	0	0	2	7.14
RIF	5	13–28	12–30	11	50.00	4	18.18	7	31.82	15	53.57	3	10.71	10	35.71
CN	10	23–48	22–51	22	100.0	0	0	0	0.00	22	78.57	5	17.86	1	3.57
S	10	17–45	19–43	19	86.36	1	4.55	2	9.10	25	89.29	1	3.57	2	7.14

AMP = ampicillin; AMS = ampicillin-sulbactam; CXM = cefuroxime; TE = tetracycline; DXT = doxycycline; CIP = ciprofloxacin; LEV = levofloxacin, SXT = trimethoprim- sulfamethoxazole; C = chloramphenicol; RIF = rifampin; CN = gentamycin; S = streptomycin

Furthermore, 24/25 (96%) *Brucella* species were resistant either to at least two antibiotics belonging to two different classes (Table 8). The same table also shows that in the class of multidrug resistant, 12/25 (48%) of *Brucella* species are resistant to at least three classes of antibiotics and classified as multidrug resistant, whereas the rest of isolates were resistant to one or two classes of antibiotics and classified as drug resistant.

**Table 8:** Multidrug resistance profile of 25 *Brucella* species.

No. of antibiotics	Antibiotic profiles	Resistant strains		No. of antibiotic classes	Resistance category
		No.	%		
5	AMP, TE, DXT, SXT, RIF	2	8%	4	Multidrug resistant
5	AMS, TE, SXT, RIF, S	2	8%	5	Multidrug resistant
5	AMS, TE, DXT, SXT, S	1	4%	4	Multidrug resistant
4	AMP, AMS, DXT, RIF	1	4%	3	Multidrug resistant
4	AMP, AMS, TE, SXT	1	4%	3	Multidrug resistant
3	AMS, SXT, RIF	3	12%	3	Multidrug resistant
3	C, RIF, CN	1	4%	3	Multidrug resistant
3	AMP, AMS, SXT	1	4%	2	Drug resistant
3	AMP, AMS, RIF	2	8%	2	Drug resistant
3	SXT, RIF, S	1	4%	3	Multidrug resistant
3	AMP, AMS, C	1	4%	2	Drug resistant
3	SXT, C, RIF	1	4%	2	Drug resistant
3	AMP, AMS, RIF	1	4%	2	Drug resistant
2	AMS, SXT	1	4%	2	Drug resistant
2	SXT, RIF	2	8%	2	Drug resistant
2	AMS, RIF	1	4%	2	Drug resistant
2	C, RIF	2	8%	2	Drug resistant
1	RIF	1	4%	1	Drug resistant

## Discussion

Brucellosis is still one of the utmost imperative zoonotic illnesses, distressing both animals and humans in many parts of the world [53]. For thousands of years, the disease has been present in the Gulf and Mediterranean regions, posing serious public health and veterinary implications [54]. The tight link between humans, foods, and cattle is the most significant component of its One-Health. Reliable identification and species determination of Brucellae obtained from animal and human origins are crucial for early management [55].

For microbial characterization and identification, Mass Assisted Laser Desorption Ionization Time of Flight Mass Spectrometry (MALDI-TOF MS) provides a quick, accurate, and cost-effective approach. Its power comes from the distinctive and unique protein profiles generated for each bacterium, which allow for reliable microbiological identification at the genus and species levels. Due to their advantages, MALDI-TOF based tests are replacing the existing techniques for conventional bacterial identification in diagnostic laboratories [56–58]. MALDI-TOF MS is a reliable approach for identifying extremely harmful microbes (*Brucella* species and *Bacillus anthracis*), including that could have been utilized as biological weapons agents, due to its high resolution and accuracy [29, 59, 60].

These organisms are often detected with morphologic, genomic, and immunologic assays that are time-consuming, inefficient, and pose a severe danger to healthcare personnel. Early identification and characterization of the potential cause is crucial for formulating a timely and successful response in the case of bioweapons or spontaneous epidemics [61]. Although *Brucella* species have a high amount of nucleotide similarity, they differ greatly in terms of host tropism, bacterial and disease characteristics, and virulence. For a long time, the lack of variety impeded the development of molecular typing methods [61].

Furthermore, despite the fact that the MBT 2.0 basic dataset (Bruker Daltonics) is utilized for standard microbial identification in clinical microbiology, which appears to contain over 3,000 particular mass spectra from several microbial species, the database lacks spectra for several species of *Brucella*. This substantially restricts its use in high-incidence nations where *Brucella* species are regularly recovered from individuals. An augmented library comprising *Brucella* species [26, 61, 62] or a custom *Brucella* library [29] have been used by several groups to improve Brucella identification.

MALDI-TOF, a proteomics-based technology, was used in conjunction with qPCR and DNA-based technologies, to recognize different species of *Brucella* obtained from several sources in the current investigation. For the routine identification of Brucellae, MBT seemed to be a fast and consistent approach [63]. For species identification, however, DNA-based methods are still required. MALDI-TOF has emerged as a quick approach for Brucellae identification in standard diagnostic laboratories over the last two decades. To increase the accuracy of *Brucella* species identification, however, robust standard databases require a lot of strains from various species and biovars [64].

In the diagnostic laboratory, MALDI-TOF MS is replacing traditional approaches for detecting bacteria. Several of the obstacles of recognizing microbial pathogens are addressed by MALDI-TOF mass spectrometry [65]. The development of libraries collecting spectra of known organisms as technology has made it feasible to identify species with similar morphological, genotypic, and biochemical traits that were formerly impossible to identify [66]. This has improved medical intervention by reducing the time it takes to diagnose illnesses caused by relatively rare species [65]. Unfortunately, there are certain drawbacks to this method. Due to intrinsic similarities among microorganisms and a limited number of spectra in the databases, improper species differentiation and mistaken identity might occur [23, 65]. These mistakes are made infrequently and may usually be avoided by performing further screening.

All novel isolates, as well as a considerable number of ancient isolated strains from the Resource Centre Libraries’ holdings in endemic regions, should really be processed and morphologically categorized to update the current database. For identifying Brucella at the genus and species, a DNA-based approaches such as qPCR is required to eliminate morphological processing and minimize the chances of research lab infection [67].

The current study additionally used a real-time PCR to confirm the MBT results. This technique offers a viable alternative to the difficult culturing and identification of *Brucella* species by means of traditional techniques [68, 69]. Real-time PCR was found to be a sensitive and specific approach for detecting and distinguishing between *B*. *abortus* and *B*. *melitensis*. This technique has the advantages of being rapid to perform, not requiring electrophoretic analysis, and not being contaminated like traditional PCR.

Because of the high cost of treating brucellosis in cattle and sheep, it is not commonly done, yet the meat of butchered animals is consumed by humans in underdeveloped nations. The most popular antibiotic combinations suggested through the WHO to cure brucellosis in humans are doxycycline with rifampicin or fluoroquinolones with rifampicin. There are few previous investigations on sensitivity testing for antimicrobials for the Brucellae, and Kirby Bauer and the E-test techniques are commonly used [33, 55].

Increased microbial resistance to traditional antibiotics has recently sparked interest in developing new antibiotic classes for the treatment of certain infectious illnesses. Only a few restricted antibiotics have clinical efficacy and good intracellular penetration for the handling of brucellosis, as stated previously by the WHO [26]. Many *B*. *melitensis* and *B*. *abortus* strains were shown to be susceptible to a wide range of antibiotics, including ceftriaxone, ciprofloxacin, levofloxacin, doxycycline, gentamycin, chloramphenicol, and streptomycin, according to our research. Trimethoprim-sulfamethoxazole (36.36%), rifampin (31.82%), ampicillin-sulbactam (27.27%), and ampicillin (22.70 percent) resistance were all found in *B*. *melitensis* isolates. Rifampin, trimethoprim-sulfamethoxazole, ampicillin, and ampicillin-sulbactam resistance were found in 35.71%, 32.14%, 32.14%, and 28.57% of *B*. *abortus* isolates, respectively.

Alamian et al. [70] found similar results when they tested 60 isolates of *B*. *melitensis* collected from blood samples against a variety of medications routinely used to treat human brucellosis. Except for 18.4% of isolates that exhibited probable resistance to ampicillin-sulbactam, nearly all strains were sensitive to ceftriaxone, doxycycline, streptomycin, trimethoprim-sulfamethoxazole, and gentamicin. Asadi et al. [71] tested 140 *B*. *melitensis* isolates against various antimicrobial agents and found that all strains were vulnerable to streptomycin, doxycycline, ciprofloxacin, moxifloxacin, and gentamicin, while only 3.5% and 35.08% of strains had moderate response to trimethoprim-sulfamethoxazole and rifampin, correspondingly.

Qadri and Ueno [72] indicated that several antimicrobial drugs such as doxycycline, ciprofloxacin, tetracycline, gentamicin, streptomycin, trimethoprim-sulfamethoxazole, and levofloxacin were all effective against *Brucella* strains collected from Saudi Arabia. Nonetheless, Khan et al. [33] observed that *B*. *melitensis* strains of animal sources were resistant to ciprofloxacin, rifampicin, and streptomycin in 75.2%, 66.7%, and 4.8%, correspondingly. Furthermore, Liu et al. [47] found that all 85 *B*. *melitensis* isolates from patients suffering from Brucellosis in China were sensitive to levofloxacin, ciprofloxacin, sparfloxacin, minocycline, gentamicin tetracycline, and doxycycline. Cotrimoxazole and rifampin resistance was found in 7.0% and 1.0%, respectively, of the isolates. In another investigation, Abdel Maksoud and his colleagues [73] found that 64% of Brucella strains recovered from Egyptian patients were resistant to rifampin. Cotrimoxazole and rifampin have limited inhibitory effect against Brucella strains, according to Lopez-Merino et al. [74].

Antibiotic resistance is a major problem in many developing nations because of improper drug administration, which results in the usage of a large number of antibiotics each year. It has also been observed that Middle Eastern countries consume significantly more systemic antibacterial medicines such as broad-spectrum β-lactam antibiotics, 3^rd^ generation cephalosporins, and quinolones than other countries [70]. As a result, the current study looked at a group of antibiotics routinely used to treat brucellosis.

Rifampin, is an antibiotic used to treat brucellosis. It works by inhibiting bacterial RNA and protein production, which makes it bactericidal [75]. Because of its high intracellular transport, this antibiotic also has an in vitro inhibitory impact against several *Brucella* strains [76]. As stated by the CLSI breakpoints for slow-growing bacteria, 31.82% (7/22) of *B*. *melitensis* isolates and 35.71% (10/28) of *B*. *abortus* isolates were susceptible to rifampin in our investigation. Rifampin resistance has already been found in 64% of Egyptian field strains [73], 36.73% in Brazil [77], 9.7% in Turkey [78], and 70% in Malaysia [79].

## Conclusions

Brucellosis is still a significant global health threat, and the One-Health approach’s most important feature is the close link between individuals, foodstuffs, and animals. Throughout Saudi Arabia, Brucellosis is an endemic zoonotic disease, with *B*. *melitensis* as the most common species of bacteria. MBT has been proposed as a viable rapid 1^st^ line screening technology for *Brucella* detection in regular clinical laboratories that requires relatively little time, work, and money. Nevertheless, in order to diagnose *Brucella* at the species level, a combination of DNA-based techniques, including real-time PCR, confirmed by microfluidic electrophoresis is required. With the exception of ampicillin, rifampin, and ampicillin-sulbactam, most of the antimicrobial drugs evaluated in this investigation showed effective inhibitory effects against *B*. *melitensis* and *B*. *abortus* and could be used in treatment regimens. Due to observed resistances, caution should be exercised while prescribing ampicillin, ampicillin-sulbactam, and rifampin. Moreover, Brucella’s intracellular localization limits the number of effective antimicrobial medicines available to treat both localized and systemic brucellosis.
